# A novel mutation in human *EMD* gene and mitochondrial dysfunction in emerin knockdown cardiomyocytes

**DOI:** 10.1111/jcmm.17532

**Published:** 2022-09-15

**Authors:** Zunhui Du, Tinfang Zhu, Menglu Lin, Yangyang Bao, Jing Qiao, Gang Lv, Yinyin Xie, Qihen Li, Jinwei Quan, Cathy Xu, Yuan Xie, Lingjie Wang, Wenjie Yang, Shengyue Wang, Liqun Wu, Tong Yin, Yucai Xie

**Affiliations:** ^1^ Department of Cardiovascular Medicine, Ruijin Hospital Shanghai Jiao Tong University School of Medicine Shanghai China; ^2^ Shanghai Institute of Hematology, State Key Laboratory of Medical Genomics, National Research Center for Translational Medicine at Shanghai, Ruijin Hospital Shanghai Jiao Tong University School of Medicine Shanghai China; ^3^ Johns Hopkins University Baltimore Maryland USA; ^4^ Department of Radiology, Ruijin Hospital Shanghai Jiao Tong University School of Medicine Shanghai China

**Keywords:** emerin, Emery–Dreifuss muscular dystrophy, mitochondrial biogenesis, mitochondrial dynamics, oxidative phosphorylation, whole‐exome sequencing

## Abstract

Emerin is an inner nuclear envelope protein encoded by the *EMD* gene, mutations in which cause Emery–Dreifuss muscular dystrophy type 1 (EDMD1). Cardiac involvement has become a major threat to patients with EDMD1; however, the cardiovascular phenotype spectrums of emerinopathy and the mechanisms by which emerin regulates cardiac pathophysiology remain unclear. Here, we identified a novel nonsense mutation (c.C57G, p.Y19X) in the *EMD* gene in a Han Chinese family through high‐throughput sequencing. Two family members were found to have EDMD1 with muscle weakness and cardiac arrhythmia. Mechanistically, we first discovered that knockdown of emerin in HL‐1 or H9C2 cardiomyocytes lead to impaired mitochondrial oxidative phosphorylation capacity with downregulation of electron transport chain complex I and IV and upregulation of complex III and V. Moreover, loss of emerin in HL‐1 cells resulted in collapsed mitochondrial membrane potential, altered mitochondrial networks and downregulated multiple factors in RNA and protein level, such as PGC1α, DRP1, MFF, MFN2, which are involved in regulation of mitochondrial biogenesis, fission and fusion. Our findings suggest that targeting mitochondrial bioenergetics might be an effective strategy against cardiac disorders caused by *EMD* mutations.

## 1 INTRODUCTION

Emerin is an integral membrane protein that localizes predominantly at the inner nuclear envelope.^1^, ^2^ In 1994, human *EMD* gene encoding for emerin was first identified as the causative gene for X‐linked Emery–Dreifuss muscular dystrophy (X‐EDMD), now assigned to the subtype EDMD type 1 (EDMD1), which is classically characterized by progressive muscle dystrophy, early joint contractures and cardiomyopathy with conduction abnormalities.^3^, ^4^, ^5^ The incidence of EDMD1 is estimated as 1 in 100,000.^6^ The characteristics include a high rate of cardiac involvement like atrioventricular conduction block, atrial flutter and fibrillation, atrial standstill, or even sudden cardiac death (SCD), which may appear before left ventricle systolic dysfunction.^7^, ^8^, ^9^ Until recently, more than 100 mutations, mostly nonsense or frameshift, have been reported, 95% of which result in loss of emerin protein.^10^ However, how emerin affects the pathophysiology of cardiac disorders remains elusive.

Mitochondria are the powerhouse of cardiomyocytes and must constantly produce large amounts of ATP to maintain contractile function of the heart.^11^ To meet substantial energy requirements, the heart metabolizes various fuels to generate ATP through oxidative phosphorylation (OXPHOS), which provides approximately 95% of the cellular energy demand.^12^, ^13^ Compromised energy production efficiency has been considered as a key mechanism of pump failure, and cardiac rhythm disruption occurs due to insufficient energy supply to ion channels and transporters.^14^, ^15^ Mitochondria are also dynamic, semiautonomous organelles continuously undergoing processes essential for maintaining mitochondrial homeostasis, such as biogenesis, fission and fusion.^16^ Mitochondrial biogenesis, a type of symmetric fission that generates two healthy mitochondria, is controlled by an important transcription factor, peroxisome proliferator activator receptor γ coactivator‐1α (PGC1‐α).^17^ In failing hearts, PGC1α is downregulated, which contributes to mitochondrial dysfunction.^18^ Mitochondrial fusion and fission are crucial to mitochondrial damage repair through recombination of organelles and segregation of injured components for lysosomal degradation.^13^ There are multiple proteins regulating this antagonistic but related process, such as dynamin‐related protein 1 (DRP1), mitochondrial fission 1 protein (FIS1), mitochondrial fission factor (MFF), which promote fission, and mitofusin 1 (MFN1), MFN2, optic atrophy 1 (OPA1), which promote fusion.^19^ Mitochondrial fusion, fission and trafficking are generally termed as mitochondrial dynamics, and they are paramount not only for mitochondrial network remodelling but also for optimal mitochondrial function in physiological conditions.^20^ Thus, we sought to test whether loss of emerin leads to defective mitochondrial biogenesis or dynamics in cardiomyocytes.

Here, we investigated a family containing 11 subjects. Two family members have EDMD1 with muscle weakness and cardiac arrhythmia, along with right atrium and ventricle enlargement in one and joint contracture in the other. Whole‐exome sequencing (WES) and Sanger sequencing were used to analyse the genetic background of this family and a novel nonsense mutation (c.C57G, p.Y19X) in the *EMD* gene was identified in both affected patients. To further characterize the function of emerin in cardiomyocytes, we silenced the *EMD* gene in HL‐1 and H9C2 cells. We found that loss of emerin caused impaired oxidative phosphorylation, aberrant electron transport chain (ETC) protein expression, collapsed mitochondrial membrane potential, altered mitochondrial networks, and decreased RNA and protein level of multiple factors involved in mitochondrial biogenesis, fusion and fission. Our findings unveil that emerin deficiency contributes to mitochondrial dysfunction in cardiomyocytes.

## 2 MATERIALS AND METHODS

### 2.1 Human blood samples

**FIGURE 1:**
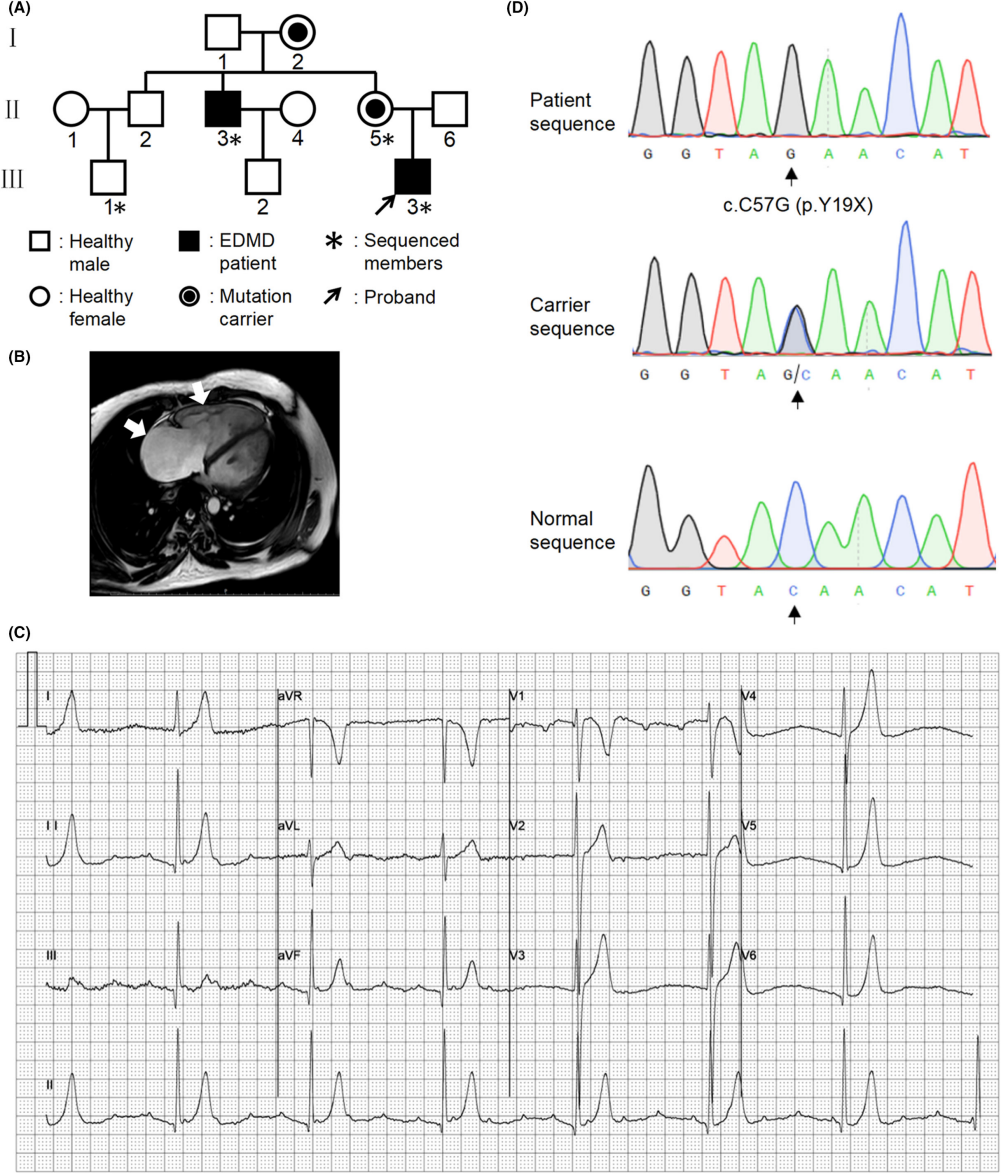
Pedigree chart, patient's clinical feature and *EMD* mutation. (A) Pedigree chart of the studied family. Genomic DNA of 4 family members was analysed (indicated with an asterisk [*]): 1 healthy individual (III‐1), 1 mutation‐carrier (II‐5) and 2 patients (II‐3, III‐3) were identified. The proband is indicated by an arrow (III‐3). (B) Cardiac enhanced MRI of the proband showing enlarged right atrium and right ventricle (indicated by white arrows). (C) The proband's ECG indicating atrial tachycardia, third degree atrioventricular block. (D) Sequence chromatogram on genomic DNA from the proband (patient sequence), II‐5 (carrier sequence) carring the c.C57G (p.Y19X) *EMD* variant, and III‐1 (normal sequence). The site of the variant is indicated by a black arrow.

We enrolled a Han Chinese family spanning three generations with 11 members (Figure 1A) from the Department of Cardiovascular Medicine in Shanghai Ruijin Hospital. Peripheral blood was collected from the affected proband and three other family members (II‐3, II‐5, III‐1, III‐3). Peripheral blood mononuclear cells were isolated and stored in liquid nitrogen until use.

### 2.2 Genetic analysis

Genomic DNA was extracted from specimens of the whole blood using QIAamp® DNA Micro Kit (Qiagen, #56304) according to the manufacturer's instructions. Sample purity was detected by Nanodrop8000 (Thermo) before database construction. Double‐terminal 2x150bp whole‐exome sequencing (WES) was performing using Illumina Novoseq 6000 (S4). The sequencing results were referenced to 1000 Genomes, gnomAD, ESP, ExAC and Kaviar databases. High‐frequency mutations were removed at a mutation frequency cut‐off of 1%, and homozygous mutations as well as synonymous mutations were also excluded. SIFT_score and Polyphen2_score were used to analyse the possible effects of mutation sites on protein sequence structure and function. Suspicious mutations were obtained according to SIFT_score ≤0.05, and Polyphen2_score ≥0.447 and ≥0.453 with reference to HumanVar database and HumanDiv database, respectively. The DAVID (https://david.ncifcrf.gov/) website was then used to annotate suspicious mutations in Disease Ontology, Gene Ontology Biological Processes, tissue distribution and KEGG pathway to further narrow down the range of suspected pathogenic genes in the family. Sanger sequencing was used to verify the suspected pathogenic *EMD* gene obtained by the above screening process. A primer pair was designed to amplify the *EMD* variant c.C57G, p.Y19X (Forward, 5’‐GACAACGATTCGGCTGTGAC‐3′; Reverse, 5′‐ CCTCTGGGTCTCGTACTCGAAG‐3′).

### 2.3 Cell culture

HL‐1 cardiomyocytes were cultured in Claycomb medium (Sigma‐Aldrich, #51800C) supplemented with 10% foetal bovine serum, 2 mmol/L L‐glutamine, 100 μmol/L norepinephrine and 100 U/ml penicillin/streptomycin in a humidified atmosphere containing 5% CO_2_ at 37°C.^21^

H9C2 cardiomyocytes were cultured in Dulbecco's modified Eagle's medium (Hyclone, #SH30243.01) supplemented with 10% foetal bovine serum and 100 U/mL penicillin/streptomycin in a humidified atmosphere containing 5% CO_2_ at 37°C.

### 2.4 Small interfering RNA transfection

The rat‐specific small interfering RNA (siRNA) targeting *EMD* was synthesized by GenePharma and used according to the manufacturer's instructions. Briefly, H9C2 cells were plated in six‐well plates. When cell confluence reached 70%–90%, *EMD* siRNA or negative control siRNA was transfected into cells using Lipofectamine™ RNAiMAX Transfection Reagent (ThermoFisher, #13778150) in serum‐free medium. After 6–12 h, the medium was replaced with complete medium and cells were then incubated at 37°C for 48–72 h for further analysis. The *EMD* siRNA forward and reverse sequences are 5′‐CUGCCUUUCUGCUCUUUGUTT‐3′ and 5′‐ACAAAGAGCAGAAAGGCAGTT‐3′, respectively.

### 2.5 Adenovirus infection

Mouse‐*EMD*‐shRNA‐EGFP adenovirus vectors were designed and purchased from HanBio Technology and infection was performed as per the manufacturer's instructions. Briefly, HL‐1 cells were plated in six‐well plates and grew to a confluence of 50%–70%. Cells were then infected with mouse‐*EMD*‐shRNA‐EGFP or EGFP‐adenovirus at a multiplicity of infection (MOI) of 50 in serum‐free medium. After 6–12 h, the medium was replaced with complete medium, and cells were incubated at 37°C for 48–72 h for further analysis. Sequence of the *EMD* shRNA is: 5′‐TCGAGGAGAGTTATTTGACCACCAAGACATTTCAAGAGAATGTCTTGGTGGTCAAATAACTCTCTTTTTTA‐3′.

### 2.6 RNA extraction and Quantitative real‐time PCR analysis

Total RNA was extracted using TRIzol (Invitrogen, #15596026) and was reverse‐transcribed to cDNA using Hifair® II 1st Strand cDNA Synthesis SuperMix (Yeason, #11123ES10). cDNA transcripts were then quantified by ABI QuantStudio™ 6 Fluorescence Quantitative PCR system with Hieff™ qPCR SYBR® Green Master Mix (Yeason, #11203ES03). The mRNA levels were normalized to 18S rRNA levels. All primer sequences were listed in Table S2.

### 2.7 Protein extraction and Western blot

Proteins were extracted from cells in RIPA buffer (Beyotime, #P0013B) with protease and phosphatase inhibitor. Cell lysates were centrifuged at 12,000 *g* for 30 min at 4°C, and the protein concentration was measured by BCA Protein Assay Kit (EpiZyme, #ZJ102). Proteins were separated by SDS‐PAGE gel and transferred to 0.45 μm PVDF membranes (Millipore, #IPVH00010), which were then blocked with 5% skim milk for 1 h at room temperature. Primary antibodies were incubated with the membrane at 4°C overnight, including anti‐emerin (1:1000 #PA5‐79201) purchased from Invitrogen; anti‐OXPHOS (1:500, #ab110413) purchased from Abcam; anti‐PGC1α (1:1000, #A12348) purchased from ABclonal Technology; anti‐DRP1 (1:1000, #8570), anti‐MFN2 (1:1000, #9482) and anti‐GAPDH (1:2000 #5174) purchased from Cell Signalling Technology. After washing thrice in TBST, membranes were incubated with horseradish peroxidase (HRP)‐labelled Goat Anti‐Rabbit IgG (Beyotime, #A0208) or Goat Anti‐Mouse IgG (Beyotime, #A0216). Following addition of ECL Prime Western Blotting Detection Reagent (Amersham Bioscience, #RPN2232), immunoblot signal was detected by GeneGnome XRQ Chemiluminescence Imaging System (Syngene). Intensity of bands was quantified using ImageJ software.

### 2.8 Mitochondrial oxygen consumption rate measurement

Mitochondrial oxygen consumption rate (OCR) was measured using Seahorse XFe96 extracellular flux analyser (Agilent Technologies). Forty eight hours after transfection, HL‐1 and H9C2 cells were plated on Seahorse XF Cell Culture Microplate (Agilent Technologies) at a density of 10^4^ cells per well and grew at 37°C with 5% CO_2_ overnight. An hour before assay, the medium was replaced by Seahorse XF DMEM Base Medium (Agilent Technologies) supplemented with 1 mM pyruvate, 2 mM glutamine and 10 mM glucose. Cells were then placed into a 37°C non‐CO_2_ incubator for 45 min to 1 h. OCR was continuously assayed and compounds were automatically injected into each well in the following concentration and order: 1.5 μM oligomycin, 3 μM FCCP and 0.5 μM Rotenone/Antimycin A for both cell lines. OCR values were normalized to cell number.

### 2.9 Live cell imaging

Seventy two hours after infection, HL‐1 cells were plated on Nunc™ Glass Bottom Dishes (ThermoFisher Scientific, #150680) and loaded with 150 nM TMRM (ThermoFisher Scientific, #I34361) or 500 nM MitoTracker® Red (ThermoFisher Scientific, #M22425) at 37°C for 30 min. Cells were then washed with PBS and observed using OMX ultra‐high‐resolution microscopic imaging system (GE Healthcare). Quantification of TMRM fluorescence intensity was performed using ImageJ software.

### 2.10 Mitochondrial network analysis

Images obtained from cultured HL‐1 cells were processed using the Mitochondrial Network Analysis (MiNA) ImageJ macro.^22^ In short, images were pre‐processed for sharpness and contrast enhancement, followed by converted to binary, and then skeletonized as a wireframe of lines one pixel wide. After that, all pixels within a skeleton were divided into three categories: end point pixels, slab pixels and junction pixels. Four distinct morphologies were recognized: puncta, rods, networks and ‘large and round’ structures. MiNA computes nine parameters, four of which were used: individuals (puncta, rods and large/round), networks (structures with at least one junction), mitochondrial footprint (total area of mitochondrial structures calculated prior to skeletonization), and mean branches per network (mean number of branches per network).

### 2.11 Statistical analysis

All data are shown as mean ± SD. Statistical analysis was performed using GraphPad Prism 8 software. Pairwise comparisons were calculated using unpaired two‐tailed student's *t*‐test. *p* < 0.05 was considered significantly different.

## 3 RESULTS

### 3.1 Pedigree, clinical feature and a novel *EMD* mutation

A family containing 11 subjects spanning three generations was studied. As the pedigree chart shows in Figure 1A, two family members were diagnosed with EDMD1 (II‐3, III‐3). The proband (III‐3), a 21‐year‐old man, had undergone amaurosis fugax while walking. Physical examination found muscular weakness in his extremities, but no scoliosis or joints contracture. Serum creatine kinase (CK) level was 545 IU/L (reference range 22–269 IU/L). Cardiac MRI revealed distinct enlarged right atrium and ventricle, a cardiac phenotype that has never been reported to our knowledge (Figure 1B). Electrocardiogram and 24 h Holter monitoring revealed atrial tachycardia, paroxysmal atrial flutter and fibrillation, third degree atrioventricular block (Figure 1C). Meanwhile, left ventricular ejection fraction was 62% and right ventricular ejection fraction was 55%. He was treated with permanent pacemaker implantation. One of his uncles (Figure 1A, II‐3) had contracture of his elbow and suffered bradycardia that led to a permanent cardiac pacemaker implantation at the age of 40. None of the other family members had obvious clinical symptoms.

Due to low prevalence in the general healthy population and absence of hereditary heart disease in II‐6 parents, we speculated that the genetic pattern of this family is X chromosome recessive inheritance, and the mother of the proband (II‐5) is a mutation‐carrier. Next, we used WES to investigate the pathogenic gene in this family (Figure 1A, II‐3, II‐5, III‐1, III‐3). Forty eight mutation loci were obtained by screening common mutations in patients II‐3, III‐3 and carrier II‐5, but not in healthy member III‐1. After removing synonymous mutations, there were 43 candidate mutation loci in the remaining 38 genes (Table S1). Single nucleotide polymorphism (SNP) frequencies were then selected by 1000 Genomes, gnomAD, ESP, ExAC and Kaviar databases to screen out the mutation sites with a mutation frequency less than 1%. Finally, the only suspected mutant gene *EMD* was obtained. In addition, Sanger sequencing was performed to reconfirm the validity of the variant and a nonsense mutation c.C57G, p.Y19X was found in the *EMD* gene on the X chromosome (Figure 1D). This mutation led to premature translation termination at the N‐terminus of the 19th amino acid (aa) in the *EMD* sequence. The mutated nucleotide and aa sites were shown in Figure S1. This mutation was identified in two male patients (Figure 1A, II‐3, III‐3) and one female asymptomatic carrier (II‐5), which has not been reported in existing databases. Therefore, combined with clinical features, we discovered a novel nonsense mutation (c.C57G, p.Y19X) in the *EMD* gene in an EDMD1 family.

### 3.2 Knockdown of emerin in HL‐1 and H9C2 cardiomyocytes leads to defective mitochondrial oxidative phosphorylation

**FIGURE 2:**
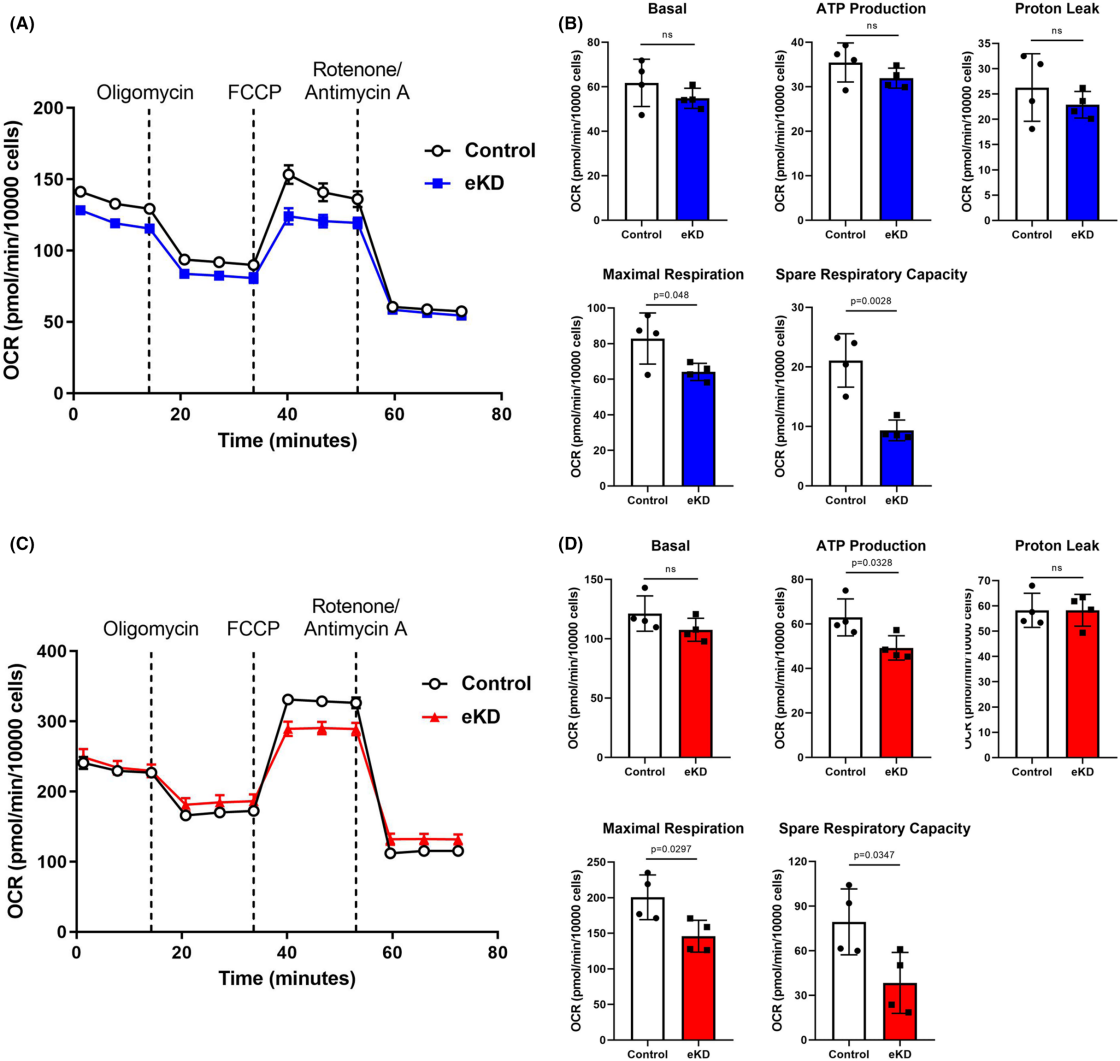
Knockdown of emerin in HL‐1 and H9C2 cardiomyocytes leads to impaired oxidative phosphorylation. (A) Representative image of sextuplicate wells demonstrated mitochondrial oxygen consumption rate (OCR) in control and eKD HL‐1 cells observed with the Seahorse multifunction energy metabolism detector. (B) Quantification of basal respiration, ATP production, proton leak, maximal respiration, spare respiratory capacity in control and eKD HL‐1 cells from four independent experiments. (C) Representative image of sextuplicate wells demonstrated mitochondrial oxygen consumption rate (OCR) in control and eKD H9C2 cells observed with the Seahorse multifunction energy metabolism detector. (D) Quantification of basal respiration, ATP production, proton leak, maximal respiration, spare respiratory capacity in control and eKD H9C2 cells from four independent experiments. FCCP: carbonyl cyanide‐4 (trifluoromethoxy) phenylhydrazone. Data show mean ± SD. Two‐tailed unpaired Student's *t*‐test. ns, not significant.

Previous studies have suggested that mitochondrial dysfunction may be involved in the pathophysiologic mechanism of cardiomyopathy and conduction disorders.^15^, ^23^ Therefore, we supposed that emerin may maintain normal electromechanical function of cardiomyocytes through mitochondria protection and sought to test our hypothesis in cultured cardiomyocytes. Next, emerin‐knockdown myocardial cell lines were chosen as experimental models for studying the function of emerin. First, the *EMD* gene was silenced in HL‐1 (derived from AT‐1 mouse atrial cardiomyocyte tumour cell line) and H9C2 (derived from embryonic BDIX rat heart) cells using adenovirus and specific siRNA, respectively. Seventy two hours later, Western blot showed that the knockdown efficiency reached 90% (Figure S2). To investigate the effect of emerin depletion in cardiomyocytes on mitochondrial bioenergetics, the Cell Mito Stress test was performed using Seahorse XF Extracellular Flux Analyzers. As shown in Figure 2A, cell oxygen consumption rate (OCR) was real‐time monitored in control and emerin knockdown (eKD) HL‐1 cells. eKD cells had similar levels of basal respiration, proton leak and ATP production compared with control cells, but the maximal respiration level and spare respiration capacity was significantly reduced (Figure 2B), indicating lower cell fitness or flexibility to demand. In keeping with this data, spare respiration capacity (%) was markedly reduced in eKD HL‐1 cells, while non‐mitochondrial oxygen consumption and coupling efficiency remained unchanged (Figure S3A). In the same way, the OXPHOS levels were tested in eKD H9C2 cells (Figure 2C). Similarly, decreased ATP production, maximal respiration level, spare respiration capacity and coupling efficiency were observed in eKD H9C2 cells, whereas other indicators were comparable to control cells (Figure 2D and Figure S3B). Taken together, these results suggested that emerin deficiency in HL‐1 and H9C2 cells impaired mitochondrial respiration capacity.

### 3.3 Knockdown of emerin in HL‐1 cells impairs mitochondrial respiratory chain integrity and membrane potential

**FIGURE 3:**
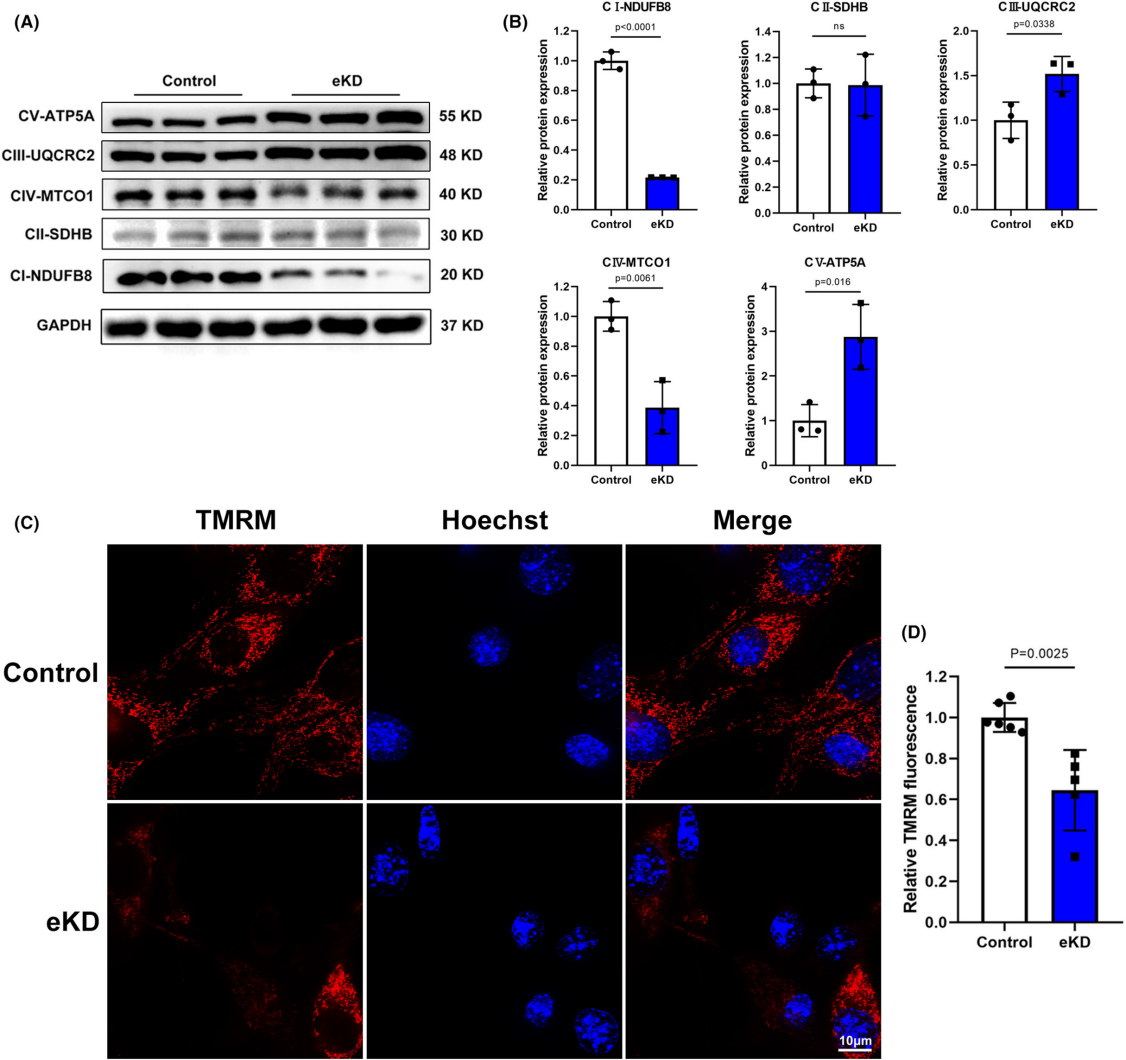
Knockdown of emerin in HL‐1 cells impairs mitochondrial respiratory chain integrity and membrane potential. (A) Western blot of mitochondrial electron transport chain complexes (I‐V) protein expression in control and eKD HL‐1 cells. GAPDH served as a loading control. (B) Quantification of CI‐NDUFB8, CII‐SDHB, CIII‐UQCRC2, CIV‐MTCO1, CV‐ATP5A protein levels normalized to GAPDH in control and eKD HL‐1 cells (*n* = 3 biological replicates per group). (C) Representative TMRM fluorescence images of control and eKD HL‐1 cells detected by OMX ultra‐high‐resolution microscopic imaging system. Scale bar: 10 μm. (D) Quantification of TMRM fluorescence intensity in control and eKD HL‐1 cells (control: *n* = 6, eKD: *n* = 5). Data show mean ± SD. Two‐tailed unpaired Student's *t*‐test. ns, not significant.

Given that healthy cardiomyocytes drive ATP production via the integrated ETC, we deduced that the defective mitochondrial respiration function shown above is due to impaired ETC complexes.^16^ With this in mind, we interrogated expression levels of protein components of ETC complexes in eKD HL‐1 cells. As predicted, significant decreased protein levels of complex I, IV components (NDUFB8, MTCO1) and increased complex III, V components (UQCRC2, ATP5A) were found in eKD HL‐1 cells compared to control cells (Figure 3A,B). As impaired ETC integrity and ATP deprivation were associated with mitochondrial membrane potential depolarization, a critical characteristic of dysfunctional mitochondria,^24^ we detected mitochondrial membrane potential in emerin‐deficient HL‐1 cells using the OMX ultra‐high‐resolution microscopic imaging system. Some of the mitochondria in eKD cells that lost their membrane potential were detected when stained with etramethylrhodamine methyl ester (TMRM) (Figure 3C,D). Collectively, these data demonstrated that emerin plays a vital role in regulating ETC complexes' expression and loss of emerin significantly decreased mitochondrial membrane potential in cardiomyocytes.

### 3.4 Mitochondrial network alterations in eKD HL‐1 cells

**FIGURE 4:**
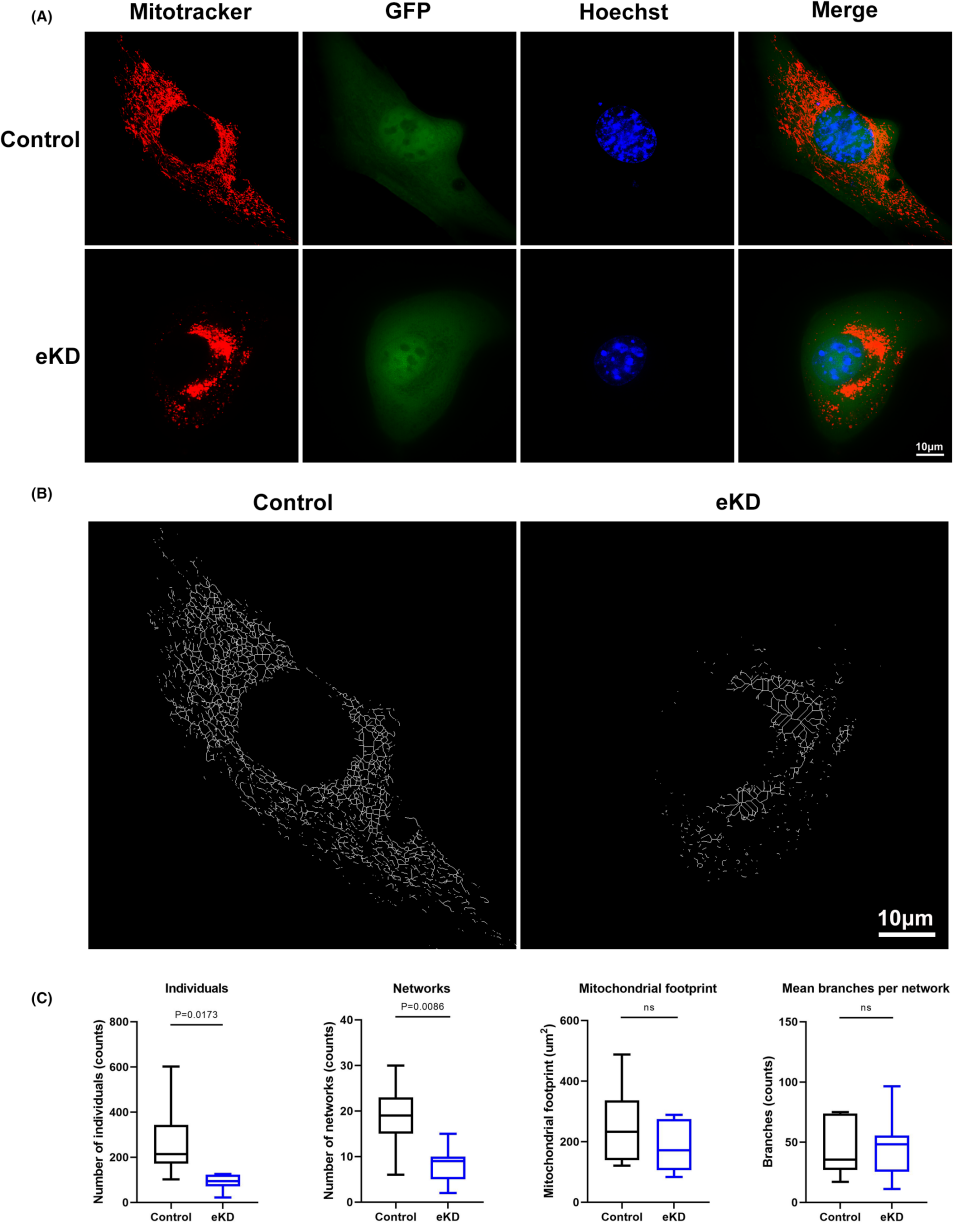
Changed mitochondrial network in emerin knockdown HL‐1 cells. (A) Representative fluorescence images showing mitochondrial morphology stained using Hoechst (blue) and Mitotracker (red) of control and eKD HL‐1 cells expressing GFP (green fluorescent protein) detected by OMX ultra‐high‐resolution microscopic imaging system. Scale bar: 10 μm. (B) The images show skeletonized mitochondrial network of control and eKD HL‐1 cells transformed from fluorescence images in A using MiNA ImageJ macro. Scale bar: 10 μm. (C) Mitochondrial network analysis of control and eKD HL‐1 cells using the MiNA ImageJ macro showing differences in: individuals, networks, mitochondrial footprint and mean number of branches per network. Seven control and 7 eKD cells from *n* = 3 independent experiments were blindly scored. Box plots show median (horizontal lines) and first to third quartile (box). Data show mean ± SD. Two‐tailed unpaired Student's *t*‐test. ns, not significant.

**FIGURE 5:**
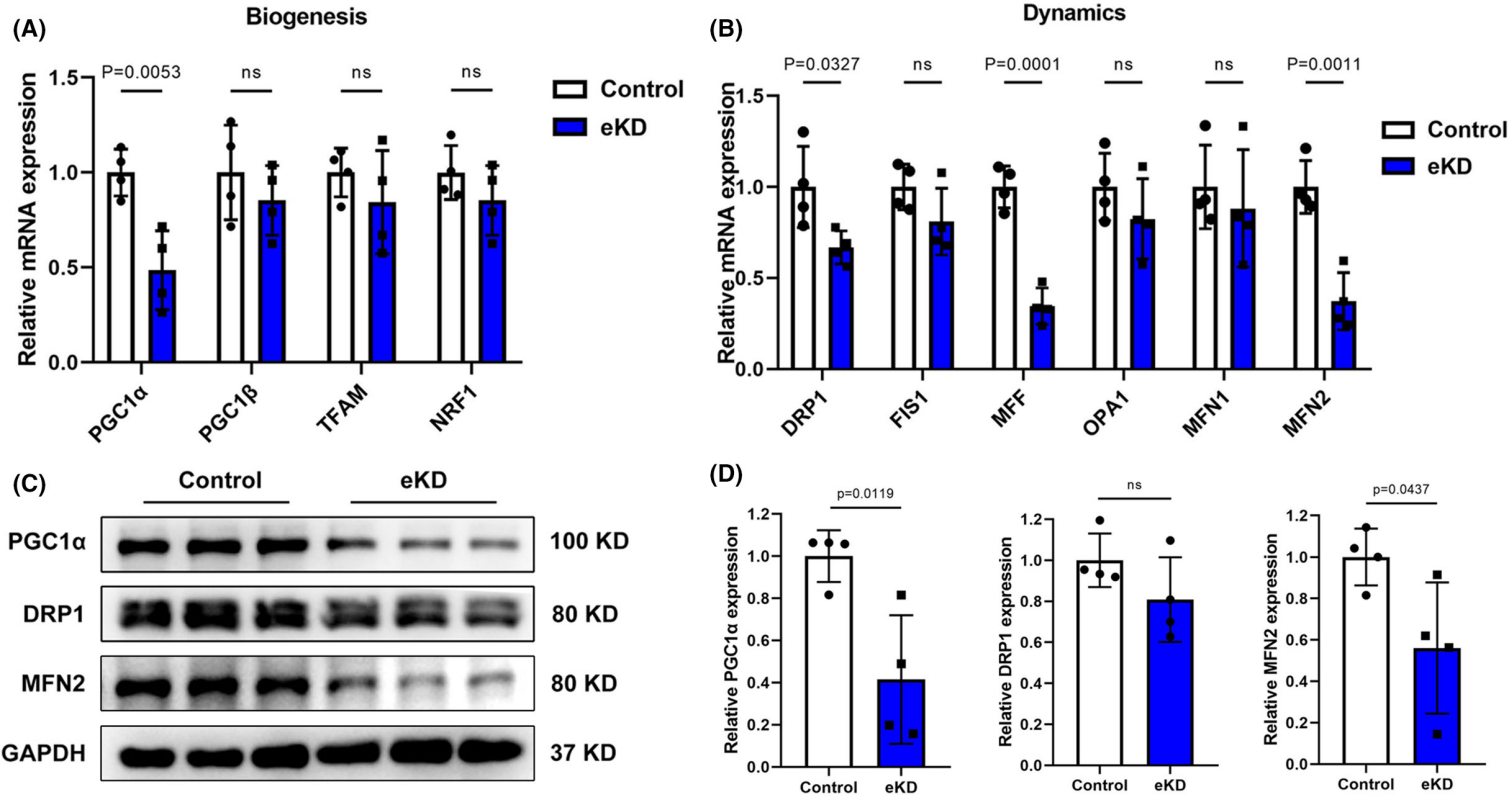
Loss of emerin in HL‐1 cells results in the decreased expression of multiple regulators involved in mitochondrial biogenesis, fission and fusion. (A) RT‐qPCR measurements of indicated genes involved in mitochondrial biogenesis in control and eKD HL‐1 cells (*n* = 4 biological replicates per group). (B) RT‐qPCR measurements of indicated genes involved in mitochondrial dynamics (fusion and fission process) in control and eKD HL‐1 cells (*n* = 4 biological replicates per group). (C) Western blot showing major proteins implicated in mitochondrial biogenesis and dynamics in control and eKD HL‐1 cells. (D) Quantification of PGC1α, DRP1, MFN2, protein expression normalized to GAPDH in control and eKD HL‐1 cells (*n* = 4 biological replicates per group). Data show mean ± SD. Two‐tailed unpaired Student's *t*‐test. ns, not significant.

In addition to mitochondrial function, mitochondrial morphology alternations have become more apparent in the pathophysiology of cardiomyopathy in recent years.^23^ To investigate the morphological and structural changes of mitochondria in eKD HL‐1 cells, we stained cells with Mitotracker Red and observed mitochondrial distribution using the OMX ultra‐high‐resolution microscopic imaging system. As representative images show in Figure 4A, eKD cells displayed distinct mitochondrial aggregation and network fragmentation compared with control cells. Decreased number of individuals and networks, but comparable mitochondrial footprint and branched networks were identified in emerin‐deficient HL‐1 cells using Mitochondrial Network Analysis (MiNA) toolset, an ImageJ macro tool for analysing cell network morphology (Figure 4B,C).^22^ To further explore the molecular mechanism underlying mitochondrial fragmentation, the expression levels of multiple genes involved in regulation of mitochondrial biogenesis, fission and fusion were assessed. As shown in Figure 5A, the master regulator of mitochondrial biogenesis, PGC1α, was significantly downregulated, while other coactivators, PPARγ coactivator‐1β (PGC1β), transcription factor A, mitochondrial (TFAM) and nuclear respiratory factor 1 (NRF1), remained unchanged. Otherwise, mitochondrial fission factors DRP1, MFF and fusion factor MFN2 were markedly downregulated (Figure 5B). These findings were partially corroborated by identification of declined levels of corresponding proteins (Figure 5C,D). Together, these observations indicated that emerin deficiency leads to reduced mitochondrial mass and altered mitochondrial morphology, which may result from impaired biogenesis and fusion.

## 4 DISCUSSION

**FIGURE 6:**
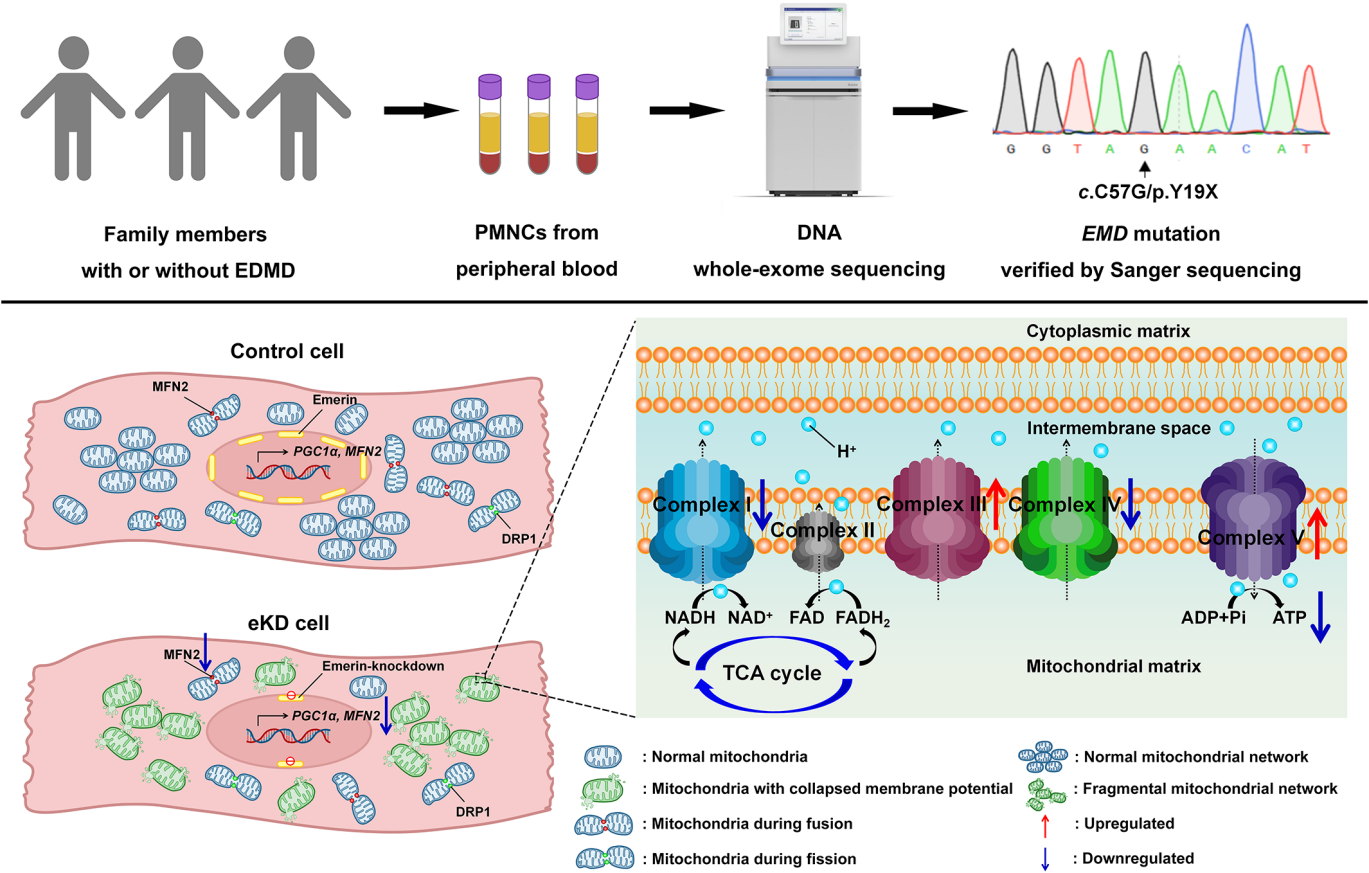
A novel mutation in human *EMD* gene and significant mitochondrial dysfunction in emerin knockdown cardiomyocytes. eKD cardiomyocytes show mitochondrial dysfunction including impaired oxidative phosphorylation, aberrant ETC protein expression, collapsed mitochondrial membrane potential, altered mitochondrial networks, decreased RNA and protein level of multiple factors involved in mitochondrial biogenesis, fusion and fission, suggesting mitochondrial bioenergetics as a potential target against cardiac disorders caused by *EMD* mutations. ETC, electron transport chain; PMNCs, peripheral mononuclear cells.

Our study discovered a novel mutation in human *EMD* gene and revealed significant mitochondrial dysfunction in cultured cardiomyocytes downregulated for emerin, as shown in Figure 6. Our genetic studies identified a novel nonsense mutation (c.C57G, p.Y19X) in the *EMD* gene in a Han Chinese family, which has not been reported in existing databases. Cardiac performance of the proband characterized by conduction defect associated with right atrium and ventricle enlargement has never been reported to our knowledge, which reflects the heterogeneity of cardiac emerinopathy. These findings expand the mutation and clinical phenotypic spectrum of EDMD1 even though the genotype–phenotype correlations remain to be investigated.

The precise molecular mechanism by which emerin mutations get involved in the pathophysiology of cardiac damage remains elusive to date. Emerin null mice show mild cardiac dysfunction; however, when under pressure overload, they present with more compromised cardiac function compared with wild‐type.^25^, ^26^ Given that mitochondria play a central role in the cardiovascular system and impaired mitochondrial bioenergetics increases the propensity to heart failure and cardiac arrhythmias, we focused on the role of mitochondrial function in cardiac emerinopathy.^15^, ^27^ In support of this idea, we observed appreciably decreased mitochondrial OXPHOS in emerin‐deficient H9C2 and HL‐1 cells, which strongly suggests the regulation of emerin on cardiac energy metabolism. Furthermore, data analysis indicated reduced ATP production, maximal respiration level and spare respiration capacity in eKD cells. These may reflect lower substrate availability, ETC integrity, and ability of cells to meet energy demands under certain stressful conditions without emerin. At the molecular level, we found a marked downregulation of ETC complex I and IV, consistent with previous research showing that disrupted complex I and IV was associated with heart failure and atrial fibrillation.^28^, ^29^, ^30^, ^31^ Otherwise, we also detected mild upregulation of complex III and V, which may serve as compensatory mechanism to sustain cellular basal oxygen consumption. Thus, we concluded that loss of emerin in cardiomyocytes leads to ETC remodelling and impaired mitochondrial bioenergetics. Combined with past in vivo data, our in vitro experiments suggested that emerin might play an important role in regulating the energy metabolism of cardiomyocytes.

Mitochondrial energy depletion induces loss of membrane potential, which subsequently causes voltage‐dependent anion channels' (VDACs) activation, Ca^2+^ overload, permeability transition pore (PTP) opening, and eventually triggering apoptosis and necrosis.^24^, ^32^ Collapsed mitochondrial membrane potential is a critical feature of various cardiovascular diseases such as heart failure, ischemia reperfusion (I/R) injury, lethal ventricular arrhythmias and SCD.^24^, ^33^, ^34^ In accordance with this, we observed decreased mitochondrial membrane potential in eKD HL‐1 cells, which may act as an early cell death signal implicated in the pathological process of cardiac emerinopathy.

Of note, cardiac mitochondrial biogenesis is an essential step in mitochondrial quality control.^35^ PGC1α, regulating not only a number of transcription factors involved in mitochondrial biogenesis, including oestrogen receptor–related α (ERRα), NRF1, but also energy metabolism, was significantly downregulated in our cell model.^36^ This is consistent with decreased mitochondrial OXPHOS capacity observed in emerin‐deficient H9C2 and HL‐1 cells. In addition, our mitochondrial network analysis revealed reduced numbers of individuals and networks, which might be partly caused by defective mitochondrial biogenesis. As such, discovering the potential mechanism linking emerin and PGC1α might facilitate reliable therapeutic targets for cardiac emerinopathy. On the other hand, mitochondria continually shape themselves via fusion and fission to meet altered energy metabolism demands.^23^ Our data displayed downregulated tendency of multiple factors involved in mitochondrial fusion and fission in eKD HL‐1 cells, suggesting a lower turnover activity. These alternations of mitochondrial dynamics might be another reason for aberrant mitochondrial morphology. Furthermore, MFN2 is a pivotal factor regulating fusion, and its protein level was significantly downregulated. In previous literature, MFN2^−/−^ mice spontaneously developed dilated cardiomyopathy with mitochondrial dysfunction and hyperfragmentation and targeting MFN2 effectively alleviated imbalanced dynamics as well as diabetic cardiomyopathy in obese diabetic mice.^37^, ^38^ Therefore, MFN2 function and interplay between fusion and fission in cardiac emerinopathy could be an interesting topic for further research.

The findings above strongly suggest that emerin has a mitochondrial protective role in cardiomyocytes; however, a few limitations could be further explored in the future. Firstly, we do not have direct evidence indicating the emerin nonsense mutant (c.C57G, p.Y19X) leads to emerin deficiency, even if the truncated forms of emerin (aa 3–41 and 3–44) had been proved undetectable in cells,^39^ and the nonsense mutation site we found giving rise to premature translation termination (located at the codon for residue 19, ahead of the 41th aa). Secondly, our experiments in vitro were based on two cardiac cell lines, which may not be able to fully retain metabolic characteristics in vivo.^40^ Neonatal or adult mouse cardiomyocytes of emerin lacking or mutant mice with mature mitochondrial networks and dynamics could be a better choice. Thirdly, our findings did not uncover the potential mechanism linking emerin and mitochondrial function. Previous studies have shown that emerin regulates multiple signal pathways such as MAPK/ERK, MKL1‐SRF, wnt/β‐catenin, notch signalling, which may underlie cardiac defects observed in EDMD1.^26^, ^41^, ^42^, ^43^ These pathways correlate closely to mitochondrial dysfunction in cardiovascular diseases.^44^, ^45^, ^46^, ^47^ Thus, a comprehensive understanding of the upstream mechanisms by which emerin regulate mitochondrial function would be a future direction of research favourable for investigating therapeutic strategies and genetic manipulations aimed to treat cardiac emerinopathy.

## 5 CONCLUSIONS

We were the first to identify a novel *EMD* nonsense mutation (c.C57G, p.Y19X) in a family associated with EDMD1. We observed that the proband manifested with distinct right atrium and ventricle enlargement accompanied with third‐degree atrioventricular block. Furthermore, we unveiled that emerin deficiency contributes to mitochondrial dysfunction in HL‐1 and H9C2 cells. Therefore, targeting mitochondrial bioenergetics may be an effective strategy against cardiac disorders caused by *EMD* mutations.

## AUTHOR CONTRIBUTIONS

**Zunhui Du:** Conceptualization (lead); data curation (lead); formal analysis (equal); investigation (equal); methodology (equal); validation (equal); visualization (equal); writing – original draft (equal); writing – review and editing (equal). **Tingfang Zhu:** Conceptualization (equal); data curation (equal); investigation (equal); methodology (equal); validation (equal); writing – original draft (equal); writing – review and editing (equal). **Menglu Lin:** Investigation (equal); validation (equal); writing – review and editing (equal). **Yangyang Bao:** Conceptualization (supporting); methodology (equal); writing – review and editing (equal). **Jing Qiao:** Methodology (equal); visualization (equal). **Gang Lv:** Methodology (equal). **Yinyin Xie:** Conceptualization (supporting); methodology (equal). **Qiheng Li:** Investigation (equal); validation (equal). **Jinwei Quan:** Investigation (equal). **Cathy Xu:** Investigation (equal). **Yuan Xie:** Writing – review and editing (equal). **Lingjie Wang:** Writing – review and editing (equal). **Wenjie Yang:** Methodology (equal). **Shengyue Wang:** Investigation (equal); methodology (equal); software (lead); visualization (equal). **Liqun Wu:** Writing – review and editing (equal). **Tong Yin:** Conceptualization (lead); funding acquisition (equal); supervision (equal); writing – review and editing (equal). **Yucai Xie:** Conceptualization (lead); data curation (lead); funding acquisition (equal); supervision (equal); writing – review and editing (equal).

## CONFLICT OF INTEREST

The authors confirm that there are no conflicts of interest.

## Supporting information


Appendix S1

## Data Availability

The data that support the findings of this study are available on request from the corresponding author. The data are not publicly available due to privacy or ethical restrictions.
