# Supplementary material for: A novel mutation in human EMD gene and mitochondrial dysfunction in emerin knockdown cardiomyocytes
Source: J Cell Mol Med. 2022 Sep 15;26(19):5054–66. doi: 10.1111/jcmm.17532 (PMC9549503; doi:10.1111/jcmm.17532)
Supplement: Supplementary file 1 — Appendix S1 [file JCMM-26-5054-s001.docx]

**A novel mutation in human *EMD* gene and mitochondrial dysfunction in emerin knockdown cardiomyocytes**

**(Supporting information)**

**Additional Figures:**


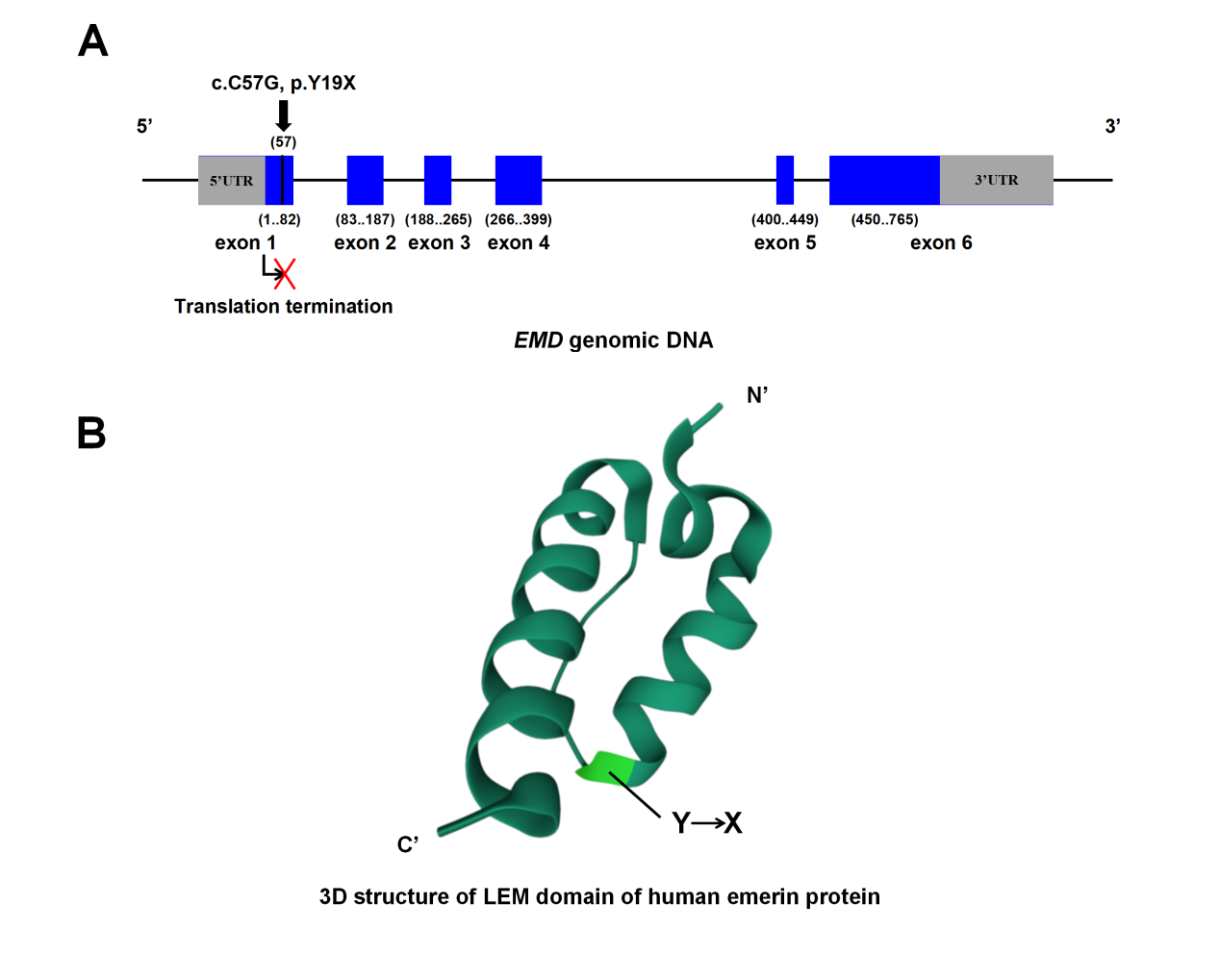


**Supplementary Figure 1** The mutated nucleotide and aa sites of the *EMD* gene. **(A)** The mutated mutated nucleotide site (c.C57G) of the *EMD* gene leading to premature translation termination at the N-terminus 57th nucleotide of the *EMD* sequence. **(B)** 3D structure of LEM domain of human emerin protein showing the mutated aa site (p.Y19X). Y: tyrosine. X: null.


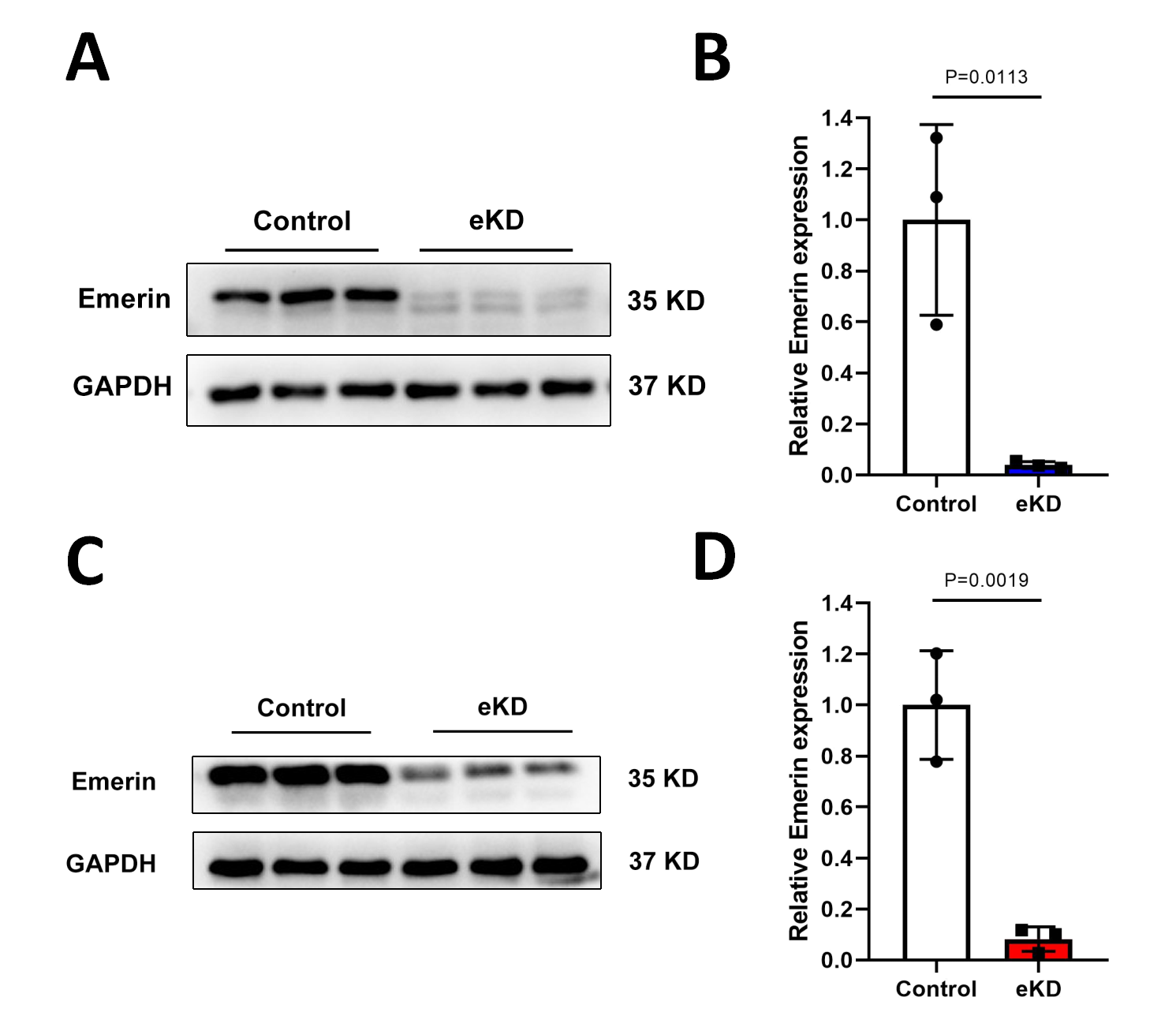


**Supplementary Figure 2** The *EMD* gene silence in HL-1 and H9C2 cells. **(A-B)** Representative western blots (A) and quantitation (B) of emerin protein levels in HL-1 cells 72 hours after the infection of adenovirus knocking down the *EMD* gene. GAPDH served as a loading control (n=3 biological replicates per group). **(C-D)** Representative western blots (C) and quantitation (D) of emerin protein levels in H9C2 cells 72 hours after the transfection of siRNA knocking down the *EMD* gene. GAPDH served as a loading control (n=3 biological replicates per group). Data show mean ± SD. Two-tailed unpaired Student’s t test.


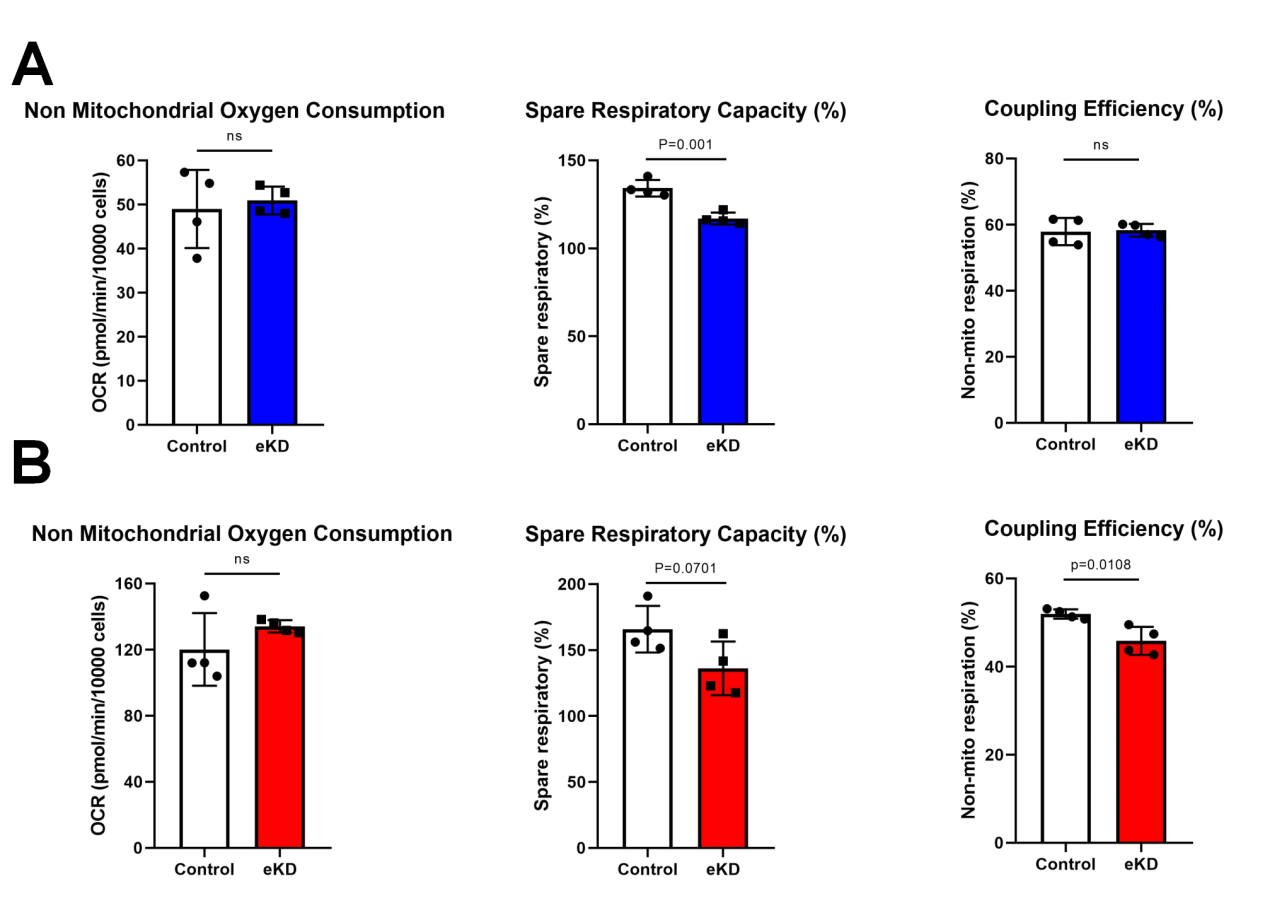


**Supplementary Figure 3** Knockdown of emerin in HL-1 and H9C2 cardiomyocytes leads to impaired oxidative phosphorylation. **(A)** Quantification of non mitochondrial oxygen consumption, spare respiratory capacity, coupling efficiency in control and eKD HL-1 cells (n=4 biological replicates per group). **(B)** Quantification of non mitochondrial oxygen consumption, spare respiratory capacity, coupling efficiency in control and eKD H9C2 cells (n=4 biological replicates per group). Data show mean ± SD. Two-tailed unpaired Student’s t test. ns: not significant.

**Additional Tables:**

**Supplementary Table 1: 38 candidate mutant gene**

| *SART1* | *IL4R* | *GDF15* | *ADD1* | *TEK* |
| --- | --- | --- | --- | --- |
| *ACADS* | *SULT1A1* | *ATP5SL* | *NUDT6* | *COL15A1* |
| *P2RX7* | *SLC12A3* | *CYBRD1* | *EDNRA* | *SLC27A4* |
| *FREM2* | *PSMD7* | *COL3A1* | *GHR* | *HTR2C* |
| *LACC1* | *MC1R* | *COMT* | *HFE* | *FMR1* |
| *CHRNA5* | *CDK5RAP3* | *ZNRF3* | *OPRM1* | *EMD* |
| *IGF1R* | *GPX4* | *ATF4* | *CHD7* |  |
| *ALG1* | *POU5F1B* | *COL6A5* | *P2RY11; PPAN-P2RY11* |  |

**Supplementary Table 2: Table of RT-qPCR primer sequences**

| Gene | Species | Forward sequence | Reverse sequence |
| --- | --- | --- | --- |
| DRP1 | Mouse | TTACGGTTCCCTAAACTTCACG | GTCACGGGCAACCTTTTACGA |
| FIS1 | Mouse | TGTCCAAGAGCACGCAATTTG | CCTCGCACATACTTTAGAGCCTT |
| MFF | Mouse | ATGCCAGTGTGATAATGCAAGT | CTCGGCTCTCTTCGCTTTG |
| MFN1 | Mouse | CCTACTGCTCCTTCTAACCCA | AGGGACGCCAATCCTGTGA |
| MFN2 | Mouse | AGAACTGGACCCGGTTACCA | CACTTCGCTGATACCCCTGA |
| NRF1 | Mouse | AGCACGGAGTGACCCAAAC | TGTACGTGGCTACATGGACCT |
| OPA1 | Mouse | CGACTTTGCCGAGGATAGCTT | CGTTGTGAACACACTGCTCTTG |
| PGC1α | Mouse | TATGGAGTGACATAGAGTGTGCT | CCACTTCAATCCACCCAGAAAG |
| PGC1β | Mouse | TCTGACGTGGACGAGCTTTCA | CCCGTCCTTCAGAGCGTCAG |
| TFAM | Mouse | GGAATGTGGAGCGTGCTAAAA | ACAAGACTGATAGACGAGGGG |
| 18s RNA | Mouse | GGACAGGATTGACAGATTGATAG | ATCGCTCCACCAACTAAGAA |
